# Management of *Helicobacter pylori* among medical doctors working in Khartoum, Sudan 2019: a cross-sectional study

**DOI:** 10.4314/ahs.v22i2.15

**Published:** 2022-06

**Authors:** Azza A Abbas, Bushra I Sulieman, Elfatih M Malik

**Affiliations:** Department of Community Medicine, Faculty of Medicine, University of Khartoum, Qasr Avenue, P.O. Box 11111, Khartoum, Sudan

**Keywords:** *Helicobacter pylori*, Medical doctor, Sudan

## Abstract

**Background:**

Various international guidelines have been developed regarding *Helicobacter pylori* (*H. pylori*) management, as it is infecting more than half of the world's population. Sudan's health system lacks guidelines regarding *H. pylori* management, leading to a discrepancy in practice. Investigating the current approach could be a step forward in the formulation of a national consensus in the management of *H. pylori*.

**Methods:**

A cross-sectional study was conducted among medical doctors currently working in Khartoum, Sudan. Participants were enrolled from platforms of medical associations through an online questionnaire. The questionnaire was scored out of 25 points, and scoring 13 or above considered a good approach. Data analysis was carried out using Statistical Package for Social Sciences (SPSS).

**Results:**

A total of 358 medical doctors participated in the study. The mean (±SD) score was 12.9(±4.5). Those who were using textbooks, campaigns, symposiums or general medical information to their primary Source of knowledge significantly scored higher. The most selected indication for both diagnosis (76.8%) and treatment (67.6%) was an active peptic ulcer. Stool antigen test (SAT) was the most preferred test (70.7%). The majority of respondents selected triple therapy (82.1%) as a first-line regimen. Only 37.7% confirmed the eradication after four weeks of stopping the treatment. They ensure eradication mainly through SAT (29%).

**Conclusion:**

A suboptimal approach was noted among medical doctors of Khartoum, Sudan, regarding *H. pylori* management. Efforts should be invested in forming national guidelines and the implementation of continuous medical education programs.

## Introduction

*Helicobacter pylori* (*H. pylori*) is a Gram-negative, micro-aerophilic, spiral, rod-shaped bacteria, infecting more than half of the world's population^1^. The prevalence of the infection in Africa is estimated to be higher than elsewhere (80%), which is probably attributable to poor hygiene and crowded living conditions^2^. In Sudan, the prevalence of infection was estimated to be 80% among patients with gastritis and Barrett's esophagus^3^ and was found to be 56.3% among Sudanese children.

*H. pylori* infection is known to be a cause of duodenal and gastric ulcers as well as gastric mucosa-associated lymphoid tissue (MALT) lymphoma^4^. The successful isolation of *H. pylori* infection in 1983^5^ from the stomachs of patients with the upper gastrointestinal disease has profoundly changed concepts of the etiology, pathogenesis, and management of the organism. This has led to the development of various guidelines regarding the management of *H. pylori*.

Numerous consensuses and guidelines aiming to disseminate the evidence-based recommendations on the management of *H. pylori* have been formulated and implemented by many organizations worldwide. The Maastricht V/Florence consensus report^6^, Kyoto global consensus report^7^, and World Gastrointestinal Organization (WGO) guidelines (*H. pylori* in developing countries)^8^ are updated guidelines for the management of *H. pylori* infection and related clinical manifestations. Adherence to these guidelines in a perspective that suits the country's situation could help avoid inadequate or unnecessary management of *H. pylori*, which might lead to serious health complications, such as gastric cancer and antibiotic resistance, respectively.

The Public Health England and National Institute for Health and Care Excellence (NICE) guidelines^9^,^10^ recommend testing patients with dyspepsia unresponsive to proton pump inhibitor (PPI), patients with a peptic ulcer history patients before NSAIDs taking, and patients with unexplained iron-deficiency anemia. They also recommend urgent endoscopy for patients above 55 years with recent onset, gastrointestinal bleeding, and unexplained non-resolving dyspepsia (over 4–6 weeks) to exclude cancer.

In 1994, *H. pylori* were classified as a class I carcinogen^11^. Infection with H. pylori leads to chronic gastritis, which can progress to intestinal metaplasia in prone patients, leading to intestinal-type gastric cancer^4^. In Sudan, stomach cancer was among the ten most common primary sites of cancer, having an incidence rate of 4 per 100 000^12^.

The increasing *H. pylori* resistance to previously efficacious antibiotic regimens, particularly to clarithromycin, and the high rate of unsuccessful eradication^13^ is of great concern and requires modification of therapeutic strategies. According to a recent meta-analysis of the WHO^14^, clarithromycin, metronidazole, and levofloxacin resistance of *H. pylori* have reached alarming points by being more than 15% worldwide. In Sudan, few studies reported H. pylori resistance to clarithromycin ranging between 7.6 to 25%^15^,^16^.

These public health hazards have forced a more proximal approach to manage the disease, generating abundant guidelines and reports. Sudan lacks national policies of management of *H. pylori* infection. This might create a considerable discrepancy in practices among clinicians, as seen in surveys from several countries, which have revealed that significant confusion still exists in the understanding of *H. pylori* infection management among primary care physicians (PCP)^17^, general practitioners^18^, trainees of internal medicine^19^ and Internists^20^.

This research is being conducted because no published research-to date- has been carried out to assess the approach of doctors towards *H. pylori* management in Sudan's hospitals. General practitioners, registrars of internal medicine, and house officers occupy most of the work spots in hospitals of Sudan, so their approaches have to be optimal regarding a common organism such as *H. pylori* . Also, Internist practices are essential to assess since no clear national guidelines are unifying the management of the infection, noting the diversity of diagnosis and treatment methods of these diseases, and the diverse backgrounds and education of Khartoum doctors. Hence, this study aims to determine medical doctors' approaches towards managing *H. pylori* infection regarding diagnosis and management and identifying sources of knowledge commonly used by medical doctors.

## Materials and Methods

### Study design

This is a cross-sectional study conducted through a non-probability convenient sampling among medical doctors currently working in Khartoum, Sudan, to assess their approaches towards managing *H. pylori* infection.

### Study population

The study included full registered specialists of gastroenterology and registrars of medicine who are currently enrolled in training. Specialists and registrars of other specialties were excluded from the study. General practitioners and house officers from various departments were enrolled in the study with inclusion criteria of previous working experience in a gastroenterology department. All participants are currently working in hospitals in Khartoum, Sudan.

Convenient sampling was executed among gastroenterology associations to recruit specialists.

Registrars, medical officers, and house officers were recruited from junior doctors' associations. An online form of a self-administered structured questionnaire was posted on these associations' platforms, and participants were asked to fill the questionnaire voluntarily. Enrollment of participants has taken time between January and September 2019.

### Sample size estimation

The sample size was estimated based on the assumption of 50% of medical doctors' malpractice management of *H. pylori*. Therefore, a sample size of 384 was estimated, having a confidence level of 95%, the corresponding Z statistic value is 1.96, and sampling errors (E) of 5%, calculated according to the formula Z2 P(1 − P)/E2.

### Data collection and research protocol

Maastricht V/Florence consensus report^6^, WGO Global Guideline (*Helicobacter Pylori* in Developing Countries)^8^, and Kyoto global consensus report^7^ were chosen as a reference for recommended practices. A questionnaire of 12 questions was designed to evaluate doctors' approach regarding *H. pylori* diagnosis and treatment. The questionnaire was consisting of 3 sections: (a) Socio-demographic data, (b) Diagnostic approach assessment, and (c) Treatment approach assessment. Only 6 of these questions were included in the questionnaire's scoring criteria, as five questions were related to the section (a) that was used as independent variables in the analysis process. One question was only rating the frequency of usage of diagnostic tests, which can't be included in the scoring system, as it only describes the pattern of use of these tests. The total score was 25 points. Physicians recording 13 or above marks were classified as having a “good” approach, while those scoring less than 13 were classified as having a “poor” approach.

### Data processing and statistical analysis

The collected data were entered into the Microsoft Excel database, and data analysis was carried out using Statistical Package for Social Sciences (SPSS) version 23.0 (SPSS Inc., Chicago, Illinois, USA).

Continuous variables were reported as mean ± S.D. The normality of the distribution of continuous variables was assessed using the Kolmogorov-Smirnov test (cut off at p=0.01). Categorical variables were described using frequency distributions and were presented as frequency and percentages [n (%)]. Continuous variables were defined as mean ± standard deviation (S.D.) and compared across variables using independent t-test and one-way analysis of variance (ANOVA). Post-hoc analysis was done for multiple comparisons. All tests were two-sided and considered significant at a p-value of less than 0.05.

## Results

### The scores and characteristics of participants

A total of 358 medical doctors participated in this study (93% response rate). They were divided into four groups for comparative purposes:

1. Specialists: 37 (10.3%) of participants were fully registered gastroenterologists (16 female, 21 male), with varying years of practice, mostly 5–10 years (37.8%).
2. Registrars: 59(16.4%) were registrars of internal medicine in training (25 females, 34 males), with most having less than five years of practice (64.4%).
3. General practitioners: 132(36.8%) were registered general practitioners (64 females, 68 males), with 78.8% less than five years of practice.
4. House officers: 130 (36.3%) were house officers in training (73 females, 57 males), having 100% less than five years in practice.

The scores of participants were ranging from 5 to 17 out of 25. The mean score was 12.9(± 4.5). Almost half of the participants had a good approach (56.7%), having a score of 13 or above. Gender, degree, practice setting, and years of practice have not shown a significant difference in participants' means of scores. Registrars have recorded the highest mean scores of 13.9 (±4.7), while specialists recorded the lowermost 11.9 (±3.4).

Source of knowledge has shown a significant difference in scores (p <0.001). Performing a Post hoc analysis, those who were using textbooks, campaigns, symposiums, or general medical information to their primary Source of knowledge scored significantly higher in scores than other sources of knowledge. General practitioners in Sudan tend to work more commonly in outpatient clinics affiliated with public hospitals. This explains the fact that all of the participating general practitioners selected the practice setting at public hospitals. Details are mentioned in table 1.

**Table 1:** Means of participant's scores regarding *H. pylori* management according to sociodemographics (n=358)

Variables	n (%)	Mean score (±SD)	*p* value
**Gender**			
Female	178(49.7%)	12.7(±4.3)	0.4
Male	180(50.3%)	13.1(±4.6)	
**Degree**			
Specialist	37(10.3%)	11.9(±3.4)	
Registrar	59(16.4%)	13.9(±4.7)	0.08
General Practitioner	132(36.8%)	12.5(±4.5)	
House officer	130(38.6%)	13.2(±4.5)	
**Practice setting**			
Public hospitals	358(100%)	12.9(±4.5)	
Private hospitals	59(16.5%)	12.9(±4.3)	0.9
Outpatient clinics	38(10.6%)	12.6(±5.3)	
Primary care centers	22(6.1%)	13(±4.8)	
**Years of practice**			
<5 years	282(78.8%)	13.1(±4.4)	
5–10 years	60(16.8%)	12.7(±4.4)	0.3
>10 years	16(4.5%)	11(±5)	
**Source of knowledge**			
Textbooks	296(82%)	13.4(±4.4)	
Medical journals	86(19%)	12.8(±3.9)	
Internet	125(34%)	13.5(±4.3)	<0.001
Campaigns and symposiums	80(22.3%)	14(±3.7)	
General medical information	125(34%)	13.6(±4.4)	

^a^ Totals in columns may be more than 100% because participants can choose multiple choices.

^b^ Measurements of the association were done by independent t-test and one-way ANOVA. (Significance= *p* <0.05).

### Diagnostic approach

**Figure 2:**
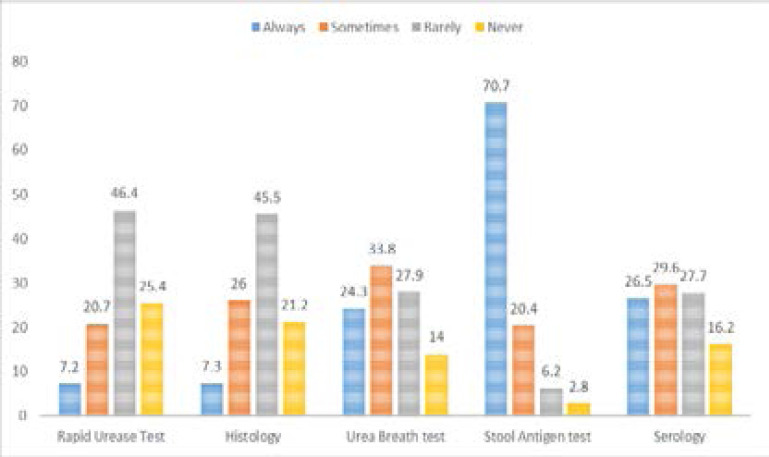
Rate of use diagnostic tests (%) (n=358), Khartoum, Sudan, 2019.

The most selected indication for diagnosis was active peptic ulcer (76.8%). Patients after resection of early gastric cancer had the lowermost selection (34.1%). 46.9% of participants selected GERD (Table 2). The stool antigen test (SAT) was the most preferred test to use regarding diagnostic tests, having 70.07% of participants rating it as “always.” The least used test was the rapid urease test (RUT), having only 7.2% of participants rating it as “always (Figure 2). Upon inquiring about discontinuing drugs before *H. pylori* testing, only 34.3% of respondents selected two weeks regarding PPI, and 33% chose four weeks in regards to antibiotics.

**Table 2:** Indications for diagnosis of *H. pylori* (n=358), Khartoum, Sudan, 2019

Scenarios	n (%)
Patient with uninvestigated dyspepsia	222(62%)
Patient with GERD	169(46.9%)
Symptomatic patient on long term PPI treatment	141(39.4%)
Patient with active peptic ulcer (gastric or duodenal)	275(76.8%)
Patient with a history of peptic ulcer (gastric or duodenal)	196(54.7%)
Patient with unexplained iron deficiency anemia	132(36.9%)
Patients who have an endoscopic appearance suggestive of gastritis on endoscopy	232(64.8%)
Patient after resection of early gastric cancer	122(34.1%)
Patient with MALT Lymphoma	154(43%)

^a^ GERD: Gastroesophageal reflux disease, PPI: Proton pump inhibitors, MALT: Mucosa-associated lymphoid-tissue.

Figure (2): Illustrates the rate of use of diagnostic tests of H. pylori among medical doctors (n=358) working in Khartoum, Sudan. Five tests (Rapid Urease test, Histology, Urea Breath test, Stool Antigen test, Serology) were compared at a scale of (always, sometimes, rarely, never).

### Treatment approach

A subset of participants (34.9%) said they would offer every patient eradication with a positive test. Active peptic ulcers scored the highest results, having 67.6% of participants choosing it, while patients after resectioning early gastric cancer re-recorded the lowest selection (13.4%) (Table 3). The majority of respondents selected triple therapy (82.1%). Quadruple therapy was much less widespread (5.3%) (Table 3). Foremost of the participants (73.2%) claimed to be confirming the eradication. Only 37.7% confirmed the eradication after four weeks of stopping the treatment. They confirm eradication mainly through SAT (29%) (Table 3).

**Table 3:** Treatment approach towards *H. pylori* (n=358), Khartoum, Sudan, 2019

Treatment indications	n(%)
Uninvestigated dyspepsia	91(25.4%)
Patient with GERD	90(25.1%)
Symptomatic patient on long term PPI treatment	74(20.7%)
Patient with active peptic ulcer (gastric or duodenal)	242(67.6%)
Patient with a history of peptic ulcer (gastric or duodenal)	114(31.4%)
Patient with unexplained iron deficiency anemia	50(14%)
Patients who have an endoscopic appearance suggestive of gastritis on endoscopy	170(47.5%)
Patient after resection of early gastric cancer	48(13.4%)
Patient with MALT lymphoma	103(28.8%)
All positive results	125(34.9%)
**First-line regimen**	
Triple therapy	294(82.1%)
Quadruple therapy	20(5.6%)
Concomitant therapy	29(8.1%)
Sequential therapy	15(4.2%)
**Time taken to confirm eradication**	
I don't perform eradication	96(26.8%)
1 week	16(4.5%)
2 weeks	85(23.7%)
3 weeks	26(7.3%)
4 weeks	135(37.7%)
**Confirmatory test**	
RUT	26(7.3%)
Histology	18(5%)
UBT	90(25.1%)
SAT	104(29%)
Serology	24(6.7%)

^a^ GERD: Gastroesophageal reflux disease, PPI: Proton pump inhibitors, MALT: Mucosa-associated lymphoid tissue, RUT: Rapid Urease test, UBT: Urea Breath test, SAT: Stool Antigen test.

^b^ Triple therapy = PPI, Clarithromycin, and Amoxicillin (or Metronidazole)

^c^ Quadruple therapy = PPI, Bismuth, Metronidazole, and Tetracycline (or doxycycline if Tetracycline is not available).

^d^ Concomitant therapy: PPI + clarithromycin+ metronidazole + amoxicillin.

^e^ Sequential therapy = PPI and amoxicillin for five days followed by PPI, Clarithromycin, and Tinidazole (or Imidazole or Metronidazole) for five days.

## Discussion

The high prevalence of *H. pylori* infection in the African continent's general population^2^, and the major complications of the untreated diseases caused by the infection^4^ made a proper approach for its management of crucial importance. This study aimed to asses the current practices of doctors regarding the management of *H. pylori* infection in Khartoum, Sudan.

Only half of the participants (56.7%) were classified as having a good approach regarding the management of *H. pylori* infection, scoring a mean of 12.9 (± 4.5) out of 25. This is a disappointing result, noting the high exposure of physicians in Sudan to patients with *H. pylori* -related diseases. Parallel inferences have been documented in different parts of the world, having 40% of participants not following the guidelines in China^21^, and lack of knowledge and adherence to guidelines was documented in Mexico^18^, the U.S.^22^, and Israel^23^.

It is worth noting that most of these countries have already constructed national guidelines and started to track the level of adherence among physicians. At the same time, Sudan still lacks unifying national policies. In a worldwide survey^24^, PCP from South America were significantly under-informed with respect to the information on the management of *H. pylori* infection when compared to PCP from the rest of the world, due to lack of regional guidelines in South America at the time of the survey. This may anticipate how non-guided management in Sudan would lead to major irregularity in practice.

Although not significant, registrars were scoring the highest. In contrast, specialists scoring the lowest may be explained by the possibility of the latter being less updated than the rest of the groups, depending more on their experience and relying less on evidence-based knowledge. This also may be related to the greater familiarity of junior physicians with international guidelines because of their more recent examinations and training. Contradictory results were noted in Iran^20^ and the U.S.^22^, with more experienced physicians recording than juniors. This could be due to the difficulty of access and the high price of validated diagnostic methods in Sudan for both specialists and junior doctors due to the country's low socioeconomic status, especially in public sectors.

The most popular Source of knowledge was textbooks (82%). In Hungary^19^, lectures and presentations were the most popular, recording 82.3%. Source of learning has shown a significant difference in the means of scores. In Iran (20), doctors who used books or educational programs recorded a significantly higher mean knowledge score compared to others, similar to our result. The practice setting has not shown a significant difference in the scores of participants (Table 1). This is dissimilar to the study of Spain^17^, where the public nature of practice showed a high level of adherence to existing guidelines. *H. pylori* testing is strongly recommended in the following conditions: active peptic ulcer, a history of peptic ulcer, dyspepsia, the endoscopic appearance of gastritis, patient with MALT lymphoma, following resection of gastric cancer, patients on long term PPI, and patients with unexplained iron deficiency anemia^6^–^8^. Overall awareness of diagnostic and treatment indications was quite concerning, having no indication recording above 76% (Table 2, 3). It might be explained that the health system in Sudan lacks resources and the relatively high cost of diagnostic procedures and treatment regimens.

This is evident upon looking at the result of 62% acknowledged testing, and only 25.4% treated the positive result of patients with dyspepsia. A similar inference was found in Pakistan^25^, with 67% of physicians investigating *H. pylori* in dyspeptic patients. This may be justified because *H. pylori* are the cause of dyspepsia in only a subset of patients^7^, so physicians tend to overlook the possibility that *H. pylori* may cause dyspepsia and referred the dyspeptic symptoms to what so-called “functional dyspepsia.”

Worth to note that the Kyoto report^7^ stated that “eradication of *H. pylori* is the first-line treatment for *H. pylori*-infected dyspeptic patients unless there are competing considerations such as comorbidities, re-infection rates in their communities, competing health priorities of society and financial cost.” This statement puts a light on the emerging issue of re-infection of *H. pylori* which is particularly high in developing countries^26^. This issue might be affecting the physicians' practice which leads to under-treatment, as the rates of re-infection are relatively high in a low socioeconomic settings.

MALT lymphoma was selected by 43% of physicians as a diagnostic indication. In contrast, only 28.8% claim to treat MALT lymphoma patients. Moreover, 64.8% of participants said they would test *H. pylori* for patients with endoscopic appearance of gastritis, while only 47.5% said they would treat the same patients. It concerns that physicians do not treat all *H. pylori*-infected patients, as only 34.9% claimed to treat every positive result. This is a costly approach with no benefit, as WGO guidelines emphasize avoiding testing the patient if not intending to treat. The rate of prescription of eradication regimen was relatively low, having active peptic ulcer scoring the highest percentage (67.6%). This is close to the figure reported in China^21^ (76%). Physicians may think that peptic ulcer patients are in good health on chronic acid suppression, but *H. pylori* eradication has been established as a cost-effective substitute for acid suppression in peptic ulcer patients^27^. Gastric cancer patients after endoscopic removal were not selected adequately among participants (34.1% for diagnosis, 13.4% for treatment). This low proportion may indicate the uncertainty of physicians regarding the carcinogenic nature of *H. pylori*.

According to guidelines, the urea breath test (UBT) is the best noninvasive test and can be replaced by SAT 6. RUT is considered the standard gold test in a patient indicated for endoscopy^8^. Serologic tests have limited clinical usefulness, and their regular performance is not recommended. Upon inquiring about each test's rate of use, SAT scored the highest (70.7% rated “always”). This can be explained by the availability and relatively low charge of the test. Histology has only recorded 7.3% of participants rating it as always. In China^21^, Histology was enormously used more than participants (89%). This contrasting preference may reflect, in part, the existing differences in the health care systems and the availability and ease of access to these tests.

Standard triple therapy is recommended as the first-line treatment for *H. pylori* unless clarithromycin resistance reached 15%^6^. In this case, quadruple therapy is recommended. Concomitant and sequential therapies are also recommended if bismuth is not available. Standard triple therapy was the most popular regimen of choice, selected by 82.1% of participants. A comparable figure was recorded in Spain^17^ regarding the use of standard triple therapy (56.4%). Quadruple therapy has the same or higher eradication rates^8^, but it was much less popular, selected only by 5.3% of participants. In China^21^, there was a much higher percentage of physicians using quadruple therapy (47%). This is most likely due to the scarce availability of bismuth in Sudan.

The majority of participants claimed that they confirm eradication (73.2%). This was lower than what was found in China (80%) and Hungary (88.1%)^19^,^21^. The most popular tests for eradication were SAT and UBT (29%, 25.1%, respectively). Pakistan^25^ figures show a resemblance concerning UBT (35%), but significantly less popularity regarding SAT (5%). 6.7% of physicians were not aware of using serological tests to confirm eradication since it doesn't differentiate between past or active infection. This is a promising result compared to Pakistani physicians^25^, where 47% confirm the eradication using serology.

PPI should be stopped two weeks before testing for *H. pylori*, while antibiotics should be stopped before four weeks^6^,^8^. This also governs testing to prove eradication. Only 37.7% of participants stated that they would confirm eradication after four weeks, indicating the low alertness that test sensitivity is affected by the antibiotics and PPI. This was also shown upon questioning the interval between the withdrawal of PPI and antibiotics before testing. Only a third of the participants recorded the correct intervals.

## Limitations

It has a selection bias as the sample was drawn conveniently. Besides, the model only reflects the physician's practices in Khartoum, Sudan, which may not be the true reflection of the whole county. Also, the questionnaire's lack of validation (although questions were based on the statements of guidelines) is considered a limitation. Nevertheless, this study might be regarded as a preliminary study, which can be improved to validate this questionnaire. However, this is the first published study of 358 physicians of Sudan, which reflects the existing diagnostic and therapeutic approaches to managing *H. pylori* in this endemic area.

The necessity of constructing practical, feasible, and evidence-based national guidelines is clarified by the inadequate practices shown in this study's results. A nation wide program must be created to remove the barriers found, and facilitate access to diagnostic methods by physicians. The efforts to educate the general practitioners and house officers about the algorithms regarding the management of *H. pylori* infection during the post-graduation period should be improved. National programs in the forms of conferences, campaigns, and symposiums would help bridge the gaps between senior and junior physicians inorder to transfer the updated knowledge and widen their horizons. Continuous assessment audits should be conducted to assess the hurdles that would face physicians in making a good approach.

## Conclusion

A suboptimal approach was noted among medical doctors of Khartoum, Sudan, regarding *H. pylori* management. The Source of knowledge has shown a significant difference in the scores of physicians. Registrars of internal medicine have recorded the highest mean scores. Efforts should be invested in forming national guidelines and the implementation of continuous medical education programs.

## Figures and Tables

**Figure 1:**
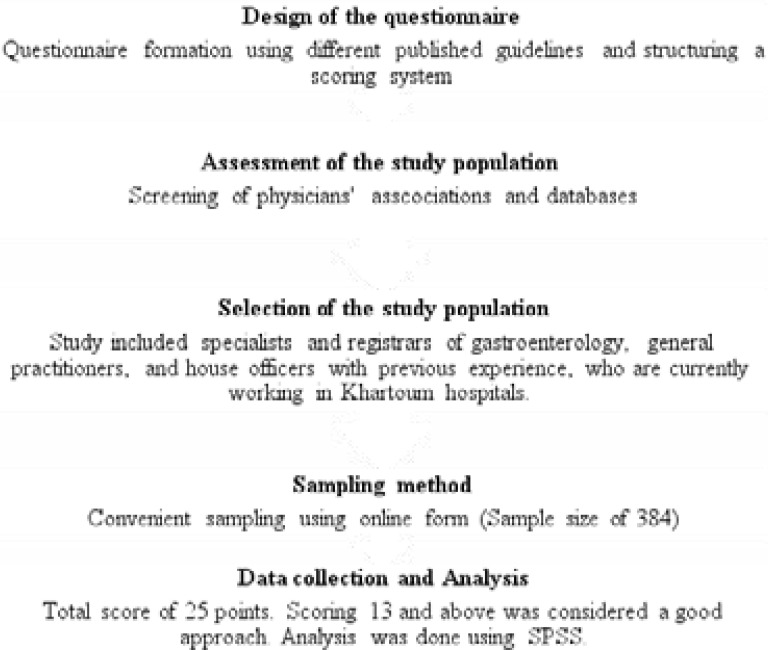
Study design flow chart, Khartoum, Sudan, 2019.

## Declaration

### Abbreviations

GERD: Gastroesophageal Reflux Disease, H. pylori: Helicobacter pylori, MALT: Mucosa-associated lymphoid tissue, NICE: National Institute for Health and Care Excellence, PCP: Primary care physicians, PPI: Proton pump inhibitor, RUT: Rapid urease test, SAT: Stool antigen test for H. pylori, UBT: Urea breath test, WGO: World Gastrointestinal Organization.
